# Catechin or quercetin guests in an intrinsically microporous polyamine (PIM-EA-TB) host: accumulation, reactivity, and release

**DOI:** 10.1039/d1ra04543a

**Published:** 2021-08-12

**Authors:** Lina Wang, Richard Malpass-Evans, Mariolino Carta, Neil B. McKeown, Shaun B. Reeksting, Frank Marken

**Affiliations:** Department of Chemistry, University of Bath Claverton Down Bath BA2 7AY UK f.marken@bath.ac.uk; EaStCHEM, School of Chemistry, University of Edinburgh Joseph Black Building, David Brewster Road Edinburgh Scotland EH9 3JF UK; Department of Chemistry, Swansea University, College of Science Grove Building, Singleton Park Swansea SA2 8PP UK; University of Bath, Materials & Chemical Characterisation Facility, MC^2^ Bath BA2 7AY UK

## Abstract

Microporous polymer materials based on molecularly “stiff” structures provide intrinsic microporosity, typical micropore sizes of 0.5 nm to 1.5 nm, and the ability to bind guest species. The polyamine PIM-EA-TB contains abundant tertiary amine sites to interact *via* hydrogen bonding to guest species in micropores. Here, quercetin and catechin are demonstrated to bind and accumulate into PIM-EA-TB. Voltammetric data suggest apparent Langmuirian binding constants for catechin of 550 (±50) × 10^3^ M^−1^ in acidic solution at pH 2 (PIM-EA-TB is protonated) and 130 (±13) × 10^3^ M^−1^ in neutral solution at pH 6 (PIM-EA-TB is not protonated). The binding capacity is typically 1 : 1 (guest : host polymer repeat unit), but higher loadings are readily achieved by host/guest co-deposition from tetrahydrofuran solution. In the rigid polymer environment, bound *ortho*-quinol guest species exhibit 2-electron 2-proton redox transformation to the corresponding quinones, but only in a thin mono-layer film close to the electrode surface. Release of guest molecules occurs depending on the level of loading and on the type of guest either spontaneously or with electrochemical stimuli.

## Abstract 2

Microporous polymer materials based on molecularly “stiff” structures provide intrinsic microporosity, typical micropore sizes of 0.5 nm to 1.5 nm, and the ability to bind guest species.

## Graphical abstract

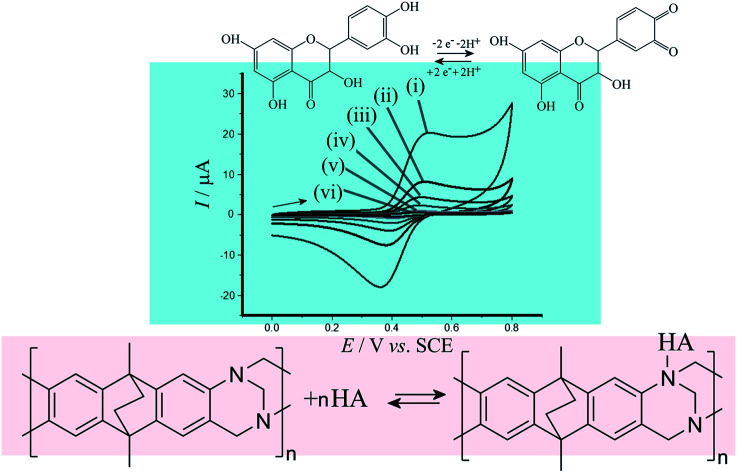

## 1. Introduction

Binding and release of guest species in microporous environments are important in selective recovery of valuable species from waste or the environment,^1^ in analysis/chromatography or in mass spectroscopy,^2^ but also for selective binding and release of drugs in medical applications.^3^ The effects of hydrogen bonding of a guest to a microporous host play an important role in modifying host–guest interactions and in pH-modulated accumulation and release processes.^4^ Here, polyamines of intrinsic microporosity are considered as hosts with rigid nanochannels to allow spontaneous accumulation and release of herbal drugs with high efficiency.

**Fig. 1:**
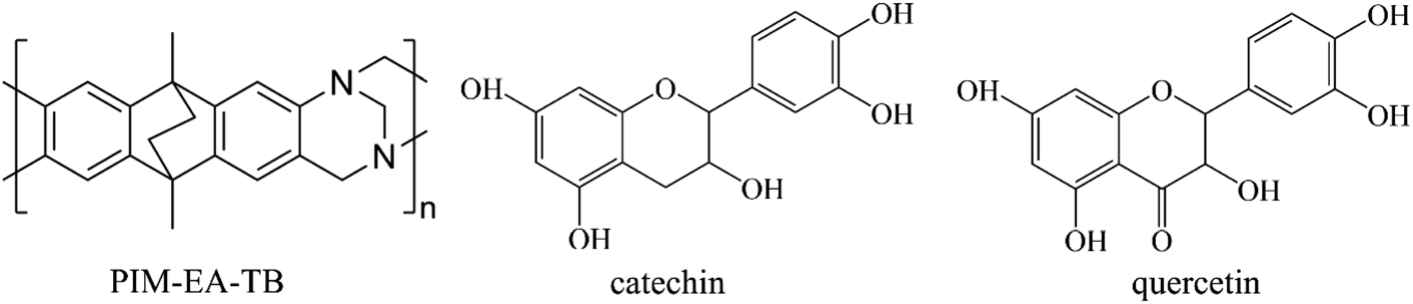
Molecular structures for PIM-EA-TB (monomeric unit m.w. 286 g mol^−1^), for the flavanol catechin (m.w. 290 g mol^−1^), and for the flavonol quercetin (m.w. 304 g mol^−1^).

Polymers of Intrinsic Microporosity (or PIMs) have emerged as a new class of processable microporous materials^5–7^ with molecular-sized voids and binding sites controlled by a rigid and contorted molecular backbone. Microporous film deposits are readily formed by solution casting. Applications of PIMs have emerged for example in the separation of gaseous species,^8^ in gas sensing,^9^ but also in condensed phase systems, for example in electrochemistry^10,11^ A prototypical PIM is the polyamine PIM-EA-TB (see molecular structure in Fig. 1) with a highly contorted and rigid backbone without any rotational freedom in single bonds, a typical molecular weight of 70 kDa, and poor packing in the solid state resulting in good solubility and processability. The internal surface area is typically 1000 m^2^ g^−1^ (based on Brunauer–Emmett–Teller analysis of nitrogen adsorption measurements^12^) and dominant pore sizes are typically in the range from 0.5 nm to 1.5 nm.

The presence of the tertiary amine functional groups in the Tröger's base (TB) structural units of PIM-EA-TB provides sites for protonation, which occurs at approximately pH 4 in aqueous environments.^13^ At solution pH values higher than 4, hydrogen bonding to these amine functional groups has been observed previously for Fe(CN)_6_^3−^ and Fe(CN)_6_^4−^ guest species^14^ and for caffeic acid.^15^ For many weakly acidic guest species, hydrogen bonding to the tertiary amine in the polymer backbone is likely to contribute to the strength of host–guest interactions and thereby likely to contribute to the rate of transport processes within PIM-EA-TB.

The binding and release of catechin and quercetin, two natural *ortho*-quinols, into/from PIM-EA-TB is investigated. Catechin is a natural flavanol and a component in herbal teas^16,17^ and in wine.^18^ For both herbal catechin^19^ and quercetin^20^ urease inhibitor effectiveness have been reported for medical applications. The electrochemistry of catechin has been carefully studied^21^ and shown to be linked to anti-oxidant characteristics^22^ and superoxide radical quenching.^23^ The formation of electrocatalytically active catechin films on glassy carbon (by electro-polymerisation) has been observed.^24^ The first deprotonation of catechin has been reported to occur at p*K*_a_ = 8.7.^25^ Quercetin appears to be slightly more acidic with an approximate p*K*_a_ = 7.1,^26^ less soluble in aqueous media, but redox-chemically very closely related to catechin. Both are *ortho*-quinols. Electrochemical properties of quercetin have been reported^27,28^ and the electrochemical detection of quercetin on carbon electrodes modified with nano-materials.^29^ Quercetin exhibits anti-oxidant properties with links to medicinal applications,^30^ and has been bound into microporous metal–organic framework ZIF-90 (ref. 31) and into chitosan^32^ or β-cyclodextrins.^33^ The release of quercetin from natural hydrogels has been studied for medicinal uses.^34^ Both catechin and quercetin contain *ortho*-quinol moieties and have been shown to form quinol-based metal complexes.^35^

In this report, voltammetric experiments are employed to explore the accumulation and reactivity of catechin and quercetin in PIM-EA-TB. The uptake of catechin or quercetin from aqueous solution is demonstrated to reach a limit close to that expected for one catechin binding to each PIM-EA-TB monomer repeat unit. Simply mixing in tetrahydrofuran (THF) solution and codeposition onto glassy carbon electrodes is demonstrated to yield the same type of deposits with the option to make films with a much higher loading. Effects of guest loading and of pH on binding and on release are reported.

## 2. Experimental

### 2.1. Chemical reagents

Catechin (≥98%, HPLC), quercetin (≥95%, HPLC), chloroform (≥99.8%), carbon (glassy, spherical powder, 2–12 μm, 99.5% trace metals basis) and phosphoric acid (85 wt%) were purchased form Sigma Aldrich. Sodium hydroxide (≥97%) and tetrahydrofuran (THF, ≥99.8%) were products of Fisher Chemical. PIM-EA-TB was synthesised following the literature method.^12^ Ultra-pure water (resistivity 18.2 MΩ cm at 20 °C) from a Thermo Scientific water purification apparatus was used to make aqueous solutions.

### 2.2. Instrumentation

Electrochemical tests were carried out with a potentiostat (Metrohm μAutolab II). Three-electrode measurements have been employed in which glassy carbon disk electrode (*Ø* 3 mm), Pt wire, and KCl-saturated calomel electrode (SCE, Radiometer REF 401) acted as the working electrode, counter electrode, and reference electrode, respectively. Phosphoric acid solution and solid sodium hydroxide were introduced to make phosphate buffer solutions with different pH values monitored with a pH meter (Jenway 3505). Qualitative and quantitative analysis of the catechin and quercetin release experiments was accomplished with liquid chromatography-mass spectroscopy (LC-MS) technique employing an Agilent 6545 Accurate-Mass Q-TOF LC/MS system. Scanning electron microscopy (SEM) images were captured with JEOL JSM-6301F FESEM equipment.

### 2.3. Procedures

#### Catechin immobilisation into PIM-EA-TB by aqueous solution uptake

PIM-EA-TB was dissolved in chloroform (or THF) solvent to form 1 mg mL^−1^ solution. One strategy of catechin immobilisation was putting PIM-EA-TB film into catechin aqueous solutions. Specifically, 2 μL of 1 mg mL^−1^ PIM-EA-TB chloroform solution was deposited on a 3 mm diameter glassy carbon disk electrode to form a film. To fulfil the uptake of catechin the electrode was immersed in catechin-containing phosphate buffer (0.1 M) solutions and left overnight (12 h) in a fridge. Then the electrode was taken out, rinsed with water, and tested in three-electrode system where 0.1 M phosphate buffer solution acted as electrolyte.

#### Catechin and quercetin immobilisation into PIM-EA-TB by codeposition of THF solutions

PIM-EA-TB, catechin, and quercetin solutions in THF were made by dissolution. PIM-EA-TB solution (1 mg mL^−1^) was mixed with catechin or quercetin solutions (with concentration of 0.1 or 1 or 10 mg mL^−1^) in a volume ratio of 1 : 1. Then 4 μL of the mixture solution was coated on a 3 mm diameter glassy carbon disk electrode, evaporated under natural condition, and ready for electrochemical performance evaluation in 0.1 M phosphate buffer solution. To enhance the surface area and currents for catechin immobilised in PIM-EA-TB, in some experiments carbon microspheres were introduced. A solution of 7.5 mg mL^−1^ PIM-EA-TB and 180 mg mL^−1^ carbon spheres in THF was prepared. Then an amount of 2 μL of the solution was deposited onto a glassy carbon electrode. The electrode was immersed into 5 μM catechin in 0.1 M PBS (pH 2) buffer overnight. For comparison, the same procedure was carried out without carbon spheres.

#### Catechin and quercetin release quantification

The same method of codeposition THF solutions of catechin or quercetin with PIM-EA-TB was adopted. Chronoamperometry was performed at different applied voltages of 0.0/−1.5/−2.0/−2.5/−3.0/−3.5 V *vs.* SCE for 5 min in 10 mM phosphate buffer solution (pH 7). The applied voltage caused alkaline conditions to develop inside of the polymer to accelerate the release process. After chronoamperometry the electrolyte solutions were analysed by LC-MS using an Agilent QTOF 6545 with Jet stream ESI spray source coupled to an Agilent 1260 Infinity II Quat pump HPLC with 1260 autosampler, column oven compartment and variable wavelength detector (VWD). The MS was operated negative ionization mode with the gas temperature at 250 °C, the drying gas at 12 L min^−1^ and the nebulizer gas at 45 psi (3.10 bar). The sheath gas temperature and flow were set to 350 °C and 12 L min^−1^, respectively. The MS was calibrated using reference calibrant introduced from the independent ESI reference sprayer. The VCap, Fragmentor and Skimmer was set to 3500, 125 and 45 respectively. Chromatographic separation of a 5 μL sample injection was performed on a InfinityLab Poroshell 120 EC-C18 (3.0 × 50 mm, 2.7 μm) using H_2_O (Merck, LC-MS grade) with 0.1% formic acid (FA, Fluka) v/v and methanol (MeOH, VWR, HiPerSolv) with 0.1% FA v/v as mobile phase A and B, respectively. The column was operated at flow rate of 0.5 mL min^−1^ at 50 °C starting with 5% mobile phase B for 0.5 min, thereafter the gradient was started for 2 min to a final 100% B, held at 100% B for 1 min then returned to 5% B for 3.9 min in a total 7.5 min run time. The VWD was set to collect 254 and 320 nm wavelengths at 2.5 Hz. Data processing was performed in Qual B 07.00 and Quant 10.0. Calibration curves for catechin and quercetin were obtained to quantify catechin or quercetin release.

## 3. Results and discussion

### 3.1. Catechin immobilisation by absorption into PIM-EA-TB

**Fig. 2:**
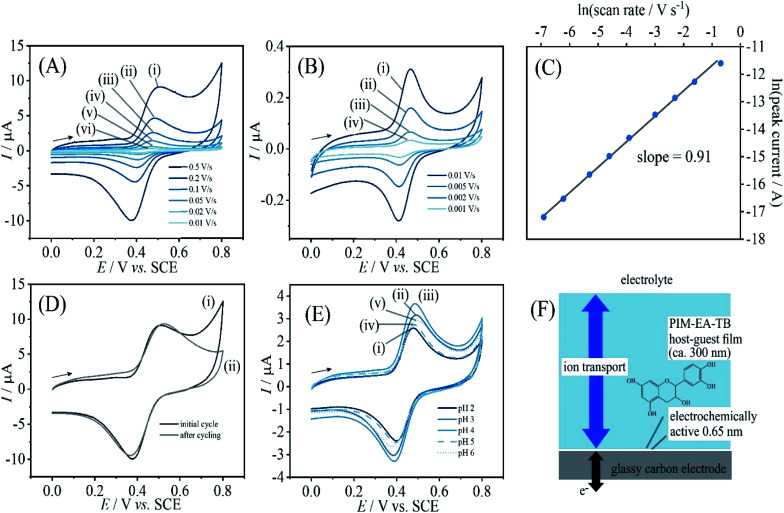
(A) Cyclic voltammograms (3 mm diameter glassy carbon electrode; scan rate (i) 0.5, (ii) 0.2, (iii) 0.1, (iv) 0.05, (v) 0.02, and (vi) 0.01 V s^−1^) for catechin bound into a PIM-EA-TB film (immobilisation in 0.1 mM catechin in 0.1 M phosphate buffer pH 2 for 12 h) and immersed into 0.1 M phosphate buffer pH 2. (B) As before, for scan rates (i) 0.01, (ii) 0.005, (iii) 0.002, and (iv) 0.001 V s^−1^. (C) Double-logarithmic plot of anodic peak current *versus* scan rate. (D) As in A(i), for the initial potential cycle (i) and the final potential cycle (ii). (E) As in A(iii), comparing the effect of pH during immobilisation (F) Schematic illustrating the thin layer redox process.

When coating a 3 mm diameter glassy carbon disk electrode with 2 μL of a solution of 1 mg mL^−1^ PIM-EA-TB, an amount of 2 μg or an in average 0.3 μm thick polymer film (assuming a density of approx. 1 g cm^−3^) was deposited. This coated electrode was then immersed into 0.1 mM catechin in 0.1 M phosphate buffer at pH 2 and left overnight (approx. 12 h) in a fridge. After rinsing with water, the electrode is employed in voltammetry experiments in 0.1 M phosphate buffer solution at pH 2. Fig. 2A and B show data from cyclic voltammetry experiments with varying potential scan rates. A well-defined oxidation–back-reduction process is observed with a midpoint potential *E*_mid_ = ½(*E*_p,ox_ + *E*_p,red_) = 0.46 V *vs.* SCE. This is consistent with literature reports for the *ortho*-quinol to *ortho*-quinone 2-electron 2-proton process for catechin^21^ (eqn (1)).1
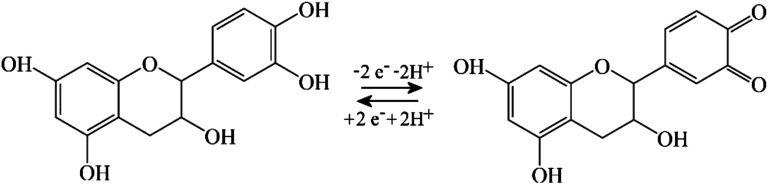


Fig. 2D demonstrates that this redox process is detected as a stable signal even after a prolonged sequence of voltammetry experiments. When varying the potential scan rate, the peak currents scale approximately linearly with scan rate (see Fig. 2C) indicative of an immobilised redox system without significant effects from diffusion on the peak shape.

Given that the peak current remains linearly related to the scan rate, it is interesting to evaluate the charge under the oxidation peak, which is here close to *Q*_p_ = 3 μC. This amount of charge corresponds to a number of 10^13^ catechin molecules or correspondingly 10^13^ PIM-EA-TB monomer units (assuming one-to-one hydrogen bonding interactions). Assuming an approximate PIM-EA-TB film density of 1 g cm^−3^, this amounts to a volume of 4.5 × 10^−9^ cm^3^ or an average thickness over the 3 mm diameter disk electrode of only 0.65 nm. This is evidence for only a monolayer of catechin molecules (within tunnel distance to the electrode; Fig. 2F) undergoing oxidation and reduction without significant propagation of charges into the bulk PIM-EA-TB film. The microporous polymer structure is too rigid to allow molecular transport/exchange for catechin on the time scale of the cyclic voltammetry experiment. Hydrogen bonds to the tertiary amine are likely to stay intact during oxidation and back-reduction of catechin although significant pH changes locally in the micropores are likely.

In order to explore effects from pH during the catechin immobilisation process, voltammetric data were obtained at pH 2 but for catechin immobilisation at pH 2, 3, 4, 5, 6, and 7 (see Fig. 2E). Perhaps surprisingly, the variation in voltammetric behaviour is very minor. A slight increase in peak current at pH 4 could be linked to higher mobility of catechin in the micropore channels of the partially protonated PIM-EA-TB host, but this is only a tentative conclusion. In order to formally express the processes during immobilisation of catechin as a function of pH equations can be suggested. The protonation of PIM-EA-TB for example in the presence of aqueous phosphate buffer is shown in eqn (2). The equilibrium constant *K*_A_ ≈ 10 000 M^−1^ is associated with an approximate p*K*_a_ of 4 under these conditions.^36^2
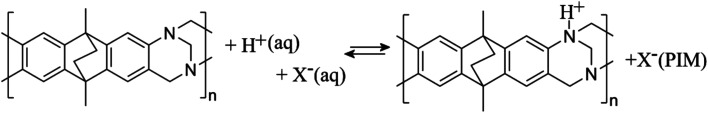


In a solution with pH > 4, the tertiary amine functional groups in PIM-EA-TB are predominantly neutral and able to bind to protons from weakly acidic guests. Catechin has a p*K*_a_ of approximately 8.7 (ref. 25) and will therefore bind *via* hydrogen bond as shown in eqn (3).3
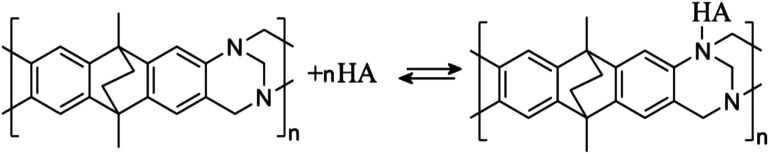


In a solution with pH < 4, the tertiary amine functional groups of PIM-EA-TB are predominantly protonated and the catechin binding process is more appropriately described as an exchange reaction in which the aqueous acid is liberated (see eqn (4)).4
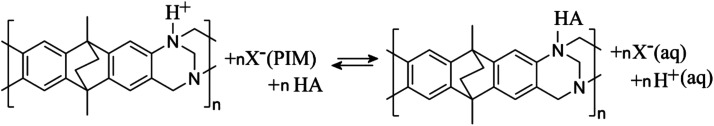


**Fig. 3:**
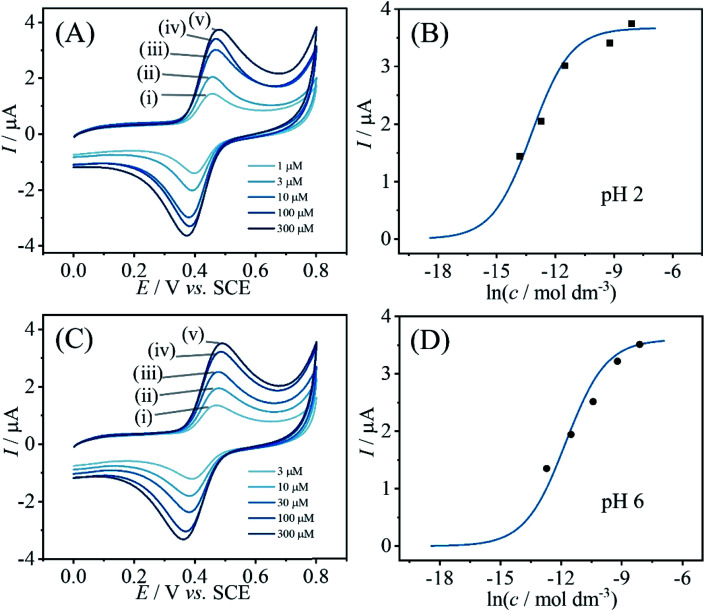
(A) Cyclic voltammograms (3 mm diameter glassy carbon electrode; scan rate 0.1 V s^−1^) for catechin bound into a PIM-EA-TB film (immobilisation in (i) 0.001, (ii) 0.003, (iii) 0.01, (iv) 0.1, (v) 0.3 mM catechin in 0.1 M phosphate buffer pH 2 for 12 h) and immersed into 0.1 M phosphate buffer pH 2. (B) Plot of peak current *versus* logarithm of catechin concentration. (C) As before, but immobilisation in (i) 0.003, (ii) 0.01, (iii) 0.03, (iv) 0.1, (v) 0.3 mM catechin in 0.1 M phosphate buffer pH 6 for 12 h. (D) Plot of peak current *versus* logarithm of catechin concentration.

Information about the apparent binding constant for catechin can be extracted from binding curves based on catechin content immobilised in PIM-EA-TB as a function of catechin concentration in solution during the immobilisation process. Fig. 3 shows data for the effect of catechin concentration on binding into PIM-EA-TB at pH = 2 (protonated PIM-EA-TB) and at pH = 6 (neutral PIM-EA-TB). Estimates for binding constants are obtained here from voltammetric peak currents plotted and fitted with a Langmuirian model (Fig. 3). The binding constant at pH 2, 550 (±50) × 10^3^ M^−1^, appears slightly higher compared to that at pH 6, 130 (±13) × 10^3^ M^−1^, but both are likely to be dominated by the hydrogen bonding interaction.

**Fig. 4:**
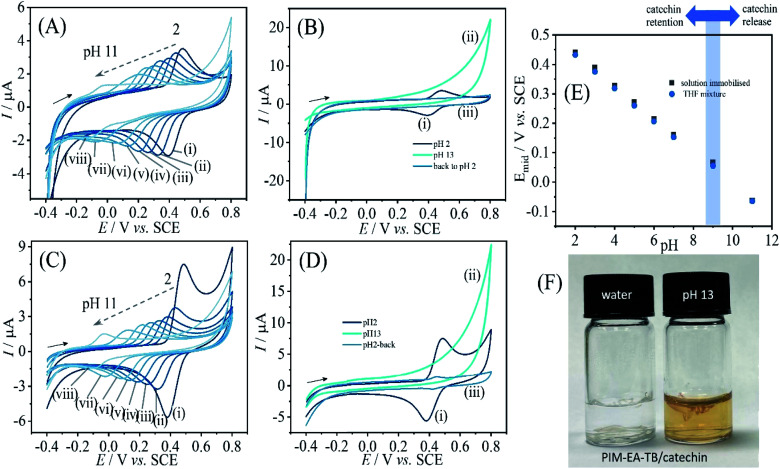
(A) Cyclic voltammograms (3 mm diameter glassy carbon electrode; scan rate 0.1 V s^−1^) for catechin bound into a PIM-EA-TB film and immersed into 0.1 M phosphate buffer at different pH values (pH = (i) 2, (ii) 3, (iii) 4, (iv) 5, (v) 6, (vi) 7, (vii) 9, and (viii) 11). (B) As before, but comparing pH 2, then pH 13, then back to pH 2. (C) Cyclic voltammograms (3 mm diameter glassy carbon electrode; scan rate 0.1 V s^−1^) for catechin co-deposited into a PIM-EA-TB film and immersed into 0.1 M phosphate buffer at different pH values. (D) As before, but comparing pH 2, then pH 13, then back to pH 2. (E) Plot of midpoint potential *E*_mid_ = ½(*E*_p,ox_ + *E*_p,red_) *versus* pH. (F) Photograph showing the PIM-EA-TB/catechin 1 : 1 film in a glass vial exposed to water and to 0.1 M phosphate buffer pH 13.

When varying the solution pH during cyclic voltammetry, well-defined Nernstian shift is observed (Fig. 4) and a gradual loss of catechin signal with every pH value. A more dramatic loss of signal occurs for pH > 9. Fig. 4B contrasts the behaviours when going from pH 2 to pH 13 and then back to pH 2. Clearly, all of the catechin has been removed under alkaline conditions. At this pH the catechin in solution can undergo deprotonation and therefore binding into the PIM-EA-TB is suppressed. Fig. 4F shows photographs for PIM-EA-TB with catechin deposited into a glass vial contrasting the behaviour under water and in pH 13 buffer solution. The colour change at pH 13 is consistent with the reported colour of catechin in alkaline solution^37^ and qualitative evidence for the release reaction (*vide infra*).

**Fig. 5:**
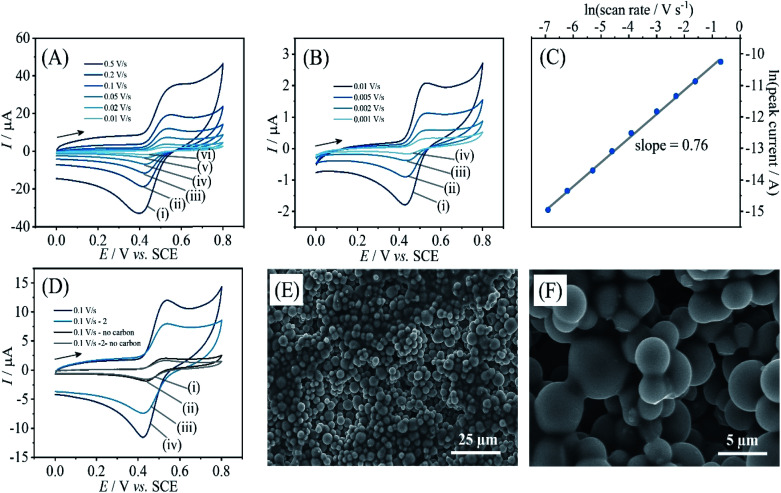
(A) Cyclic voltammograms (3 mm diameter glassy carbon electrode with 0.36 mg carbon microspheres and 15 μg PIM-EA-TB; scan rate (i) 0.5, (ii) 0.2, (iii) 0.1, (iv) 0.05, (v) 0.02, and (vi) 0.01 V s^−1^) for catechin bound into a PIM-EA-TB film (immobilisation in 0.005 mM catechin in 0.1 M phosphate buffer pH 2 for 12 h) and immersed into 0.1 M phosphate buffer pH 2. (B) As before, for scan rates (i) 0.01, (ii) 0.005, (iii) 0.002, and (iv) 0.001 V s^−1^. (C) Double-logarithmic plot of anodic peak current *versus* scan rate. (D) As in A(iii), for the initial potential cycle (i) and the second potential cycle (ii) and comparison to data obtained without carbon microspheres for the initial potential cycle (iii) and the second potential cycle (iv). (E and F) Scanning electron micrographs (SEM) of carbon microsphere/PIM-EA-TB deposits.

Due to these electrochemical processes occurring only in a very thin layer close to the electrode surface, currents remain low. It is possible to enhance currents by introducing a higher surface area. Carbon microspheres of typically 2 to 12 μm diameter (see Fig. 5E and F) can be employed and directly co-deposited with PIM-EA-TB. Here, 360 μg carbon were deposited together with 15 μg PIM-EA-TB. This is consistent with the same amount of PIM-EA-TB, but an approx. 24 times increase in total carbon surface area (assuming 6 μm diameter spheres). Fig. 5 shows data for oxidation of catechin (after binding from a 5 μM catechin solution). The peak current for catechin oxidation is approx. 2 μA at 0.01 V s^−1^ scan rate. For comparison, a current of 0.2 μA was observed for the same amount of PIM-EA-TB on a flat surface after immersion in 100 μM catechin (Fig. 2B). Therefore, the observed currents with carbon microspheres are substantially higher. A corresponding increase in capacitive background current is consistent with carbon microspheres increasing the surface area (see Fig. 5D).

### 3.2. Catechin immobilisation by codeposition with PIM-EA-TB

**Fig. 6:**
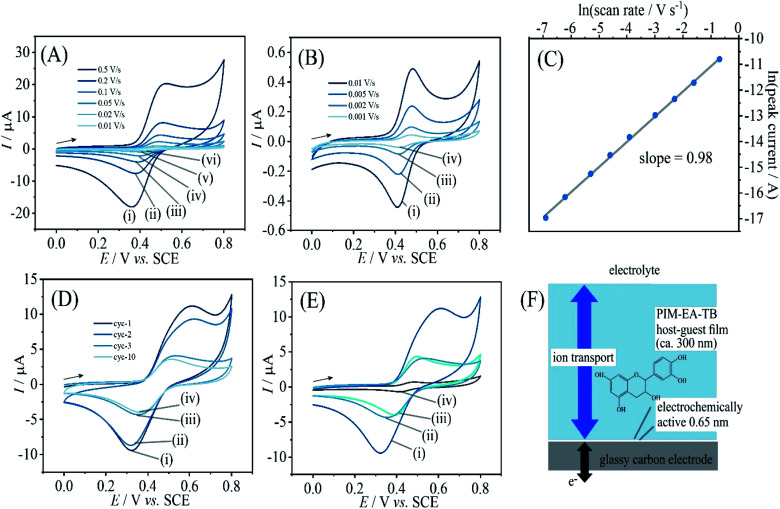
(A) Cyclic voltammograms (3 mm diameter glassy carbon electrode; scan rate (i) 0.5, (ii) 0.2, (iii) 0.1, (iv) 0.05, (v) 0.02, and (vi) 0.01 V s^−1^) for catechin co-deposited with PIM-EA-TB into a film (2 μg catechin with 2 μg PIM-EA-TB) and immersed into 0.1 M phosphate buffer pH 2. (B) As before, for scan rates (i) 0.01, (ii) 0.005, (iii) 0.002, and (iv) 0.001 V s^−1^. (C) Double-logarithmic plot of anodic peak current *versus* scan rate. (D) As in A(i), for 20 μg catechin with 2 μg PIM-EA-TB showing potential cycles 1, 2, 3, and 10. (E) As in A(i), comparing (i) 20 μg catechin/2 μg PIM-EA-TB cycle 1, (ii) 20 μg catechin/2 μg PIM-EA-TB cycle 3, (iii) 2 μg catechin/2 μg PIM-EA-TB cycle 1, and (iv) 0.2 μg catechin/2 μg PIM-EA-TB cycle 1. (F) Schematic illustrating the thin layer redox process.

PIM-EA-TB is a highly processable polymer and dissolves into tetrahydrofuran (THF) at low concentrations. Solutions of PIM-EA-TB and catechin in THF can be combined and deposited directly onto the glassy carbon electrode. Fig. 6 shows cyclic voltammetry data for 2 μg catechin codeposited with 2 μg PIM-EA-TB. Given the molecular weights of the PIM-EA-TB repeat unit and of catechin, this corresponds approximately to a 1 : 1 molecular ratio and should lead to effective hydrogen binding. Data in Fig. 6A and B are very similar to data in Fig. 2A and B. This confirms that 1 : 1 polymer host repeat unit : guest complexes are formed during catechin absorption, but it also confirms that the codeposition method is viable. Fig. 6C demonstrates that again a linear increase in peak current with scan rate is observed. Only a very thin layer at the electrode surface is electrochemically active (Fig. 6F).

Fig. 6D shows voltammetry data for the case of 1 : 10 polymer repeat unit : catechin ratio. The catechin oxidation peak current seems higher, but very rapidly drops to the value observed for 1 : 1 polymer repeat unit : catechin complexes. Therefore, catechin is readily leaching out to leave only the bound catechin in the microporous structure. Fig. 6E shows data also for the 1 : 0.1 host monomer : catechin codeposit which results in a considerably lower but stable current response. The effect of solution pH during voltammetric experiments is very similar for PIM-EA-TB films with catechin adsorbed from solution (Fig. 2A and B) and for catechin codeposited in 1 : 1 ratio (Fig. 6A and B).

### 3.3. Quercetin immobilisation by codeposition with PIM-EA-TB

**Fig. 7:**
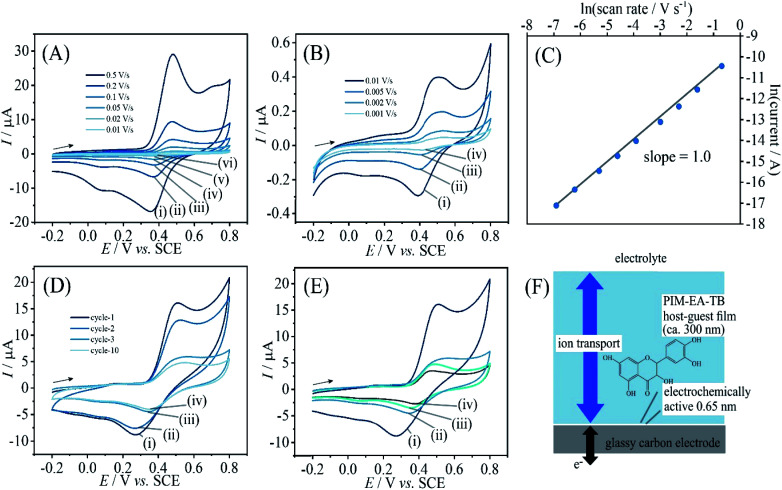
(A) Cyclic voltammograms (3 mm diameter glassy carbon electrode; scan rate (i) 0.5, (ii) 0.2, (iii) 0.1, (iv) 0.05, (v) 0.02, and (vi) 0.01 V s^−1^) for quercetin co-deposited with PIM-EA-TB into a film (2 μg quercetin with 2 μg PIM-EA-TB) and immersed into 0.1 M phosphate buffer pH 2. (B) As before, for scan rates (i) 0.01, (ii) 0.005, (iii) 0.002, and (iv) 0.001 V s^−1^. (C) Double-logarithmic plot of anodic peak current *versus* scan rate. (D) As in A(i), for 20 μg quercetin with 2 μg PIM-EA-TB showing potential cycles 1, 2, 3, and 10. (E) As in A(i), comparing (i) 20 μg quercetin/2 μg PIM-EA-TB cycle 1, (ii) 20 μg quercetin/2 μg PIM-EA-TB cycle 3, (iii) 2 μg quercetin/2 μg PIM-EA-TB cycle 1, and (iv) 0.2 μg quercetin/2 μg PIM-EA-TB cycle 1. (F) Schematic illustrating the thin layer redox process.

Quercetin is significantly less soluble in water when compared to catechin. Therefore, experiments for adsorption into PIM-EA-TB have not been performed. However, codeposition of the 1 : 1 polymer repeat unit : quercetin films is readily achieved and the voltammetric behaviour can be investigated. Data in Fig. 7A and B show oxidation and back-reduction peaks for quercetin in PIM-EA-TB immersed in pH 2 phosphate buffer solution. Oxidation (see eqn (5)) and reduction follow the 2-electron 2-proton mechanism with a midpoint potential consistent with that reported in the literature.^27,28^ Peak currents exhibit a linear relationship to scan rate consistent with a thin film of redox-active molecules close to the electrode surface. The charge under voltametric peaks is very similar to that observed for catechin and therefore a very similar redox active film (probably within tunnelling distance, Fig. 7F) can be assumed.5
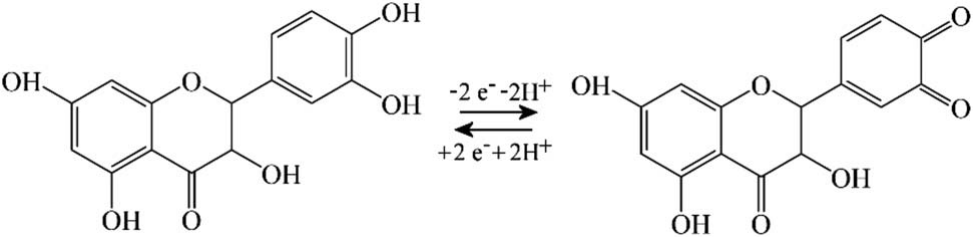


Secondary peaks observed at higher scan rate (see Fig. 7A) could be linked to localised pH imbalances in the microporous host, but could also be associated with subsequent oxidation steps as reported by Brett *et al.*^28^ Data in Fig. 7D and E suggest that (similar to the case of catechin) excess quercetin (1 : 10 host polymer repeat unit : quercetin) can be leaching out. A stable signal under cyclic voltammetry conditions is observed after 10 potential cycles and this is consistent with that for the 1 : 1 host polymer repeat unit : quercetin film deposit.

**Fig. 8:**
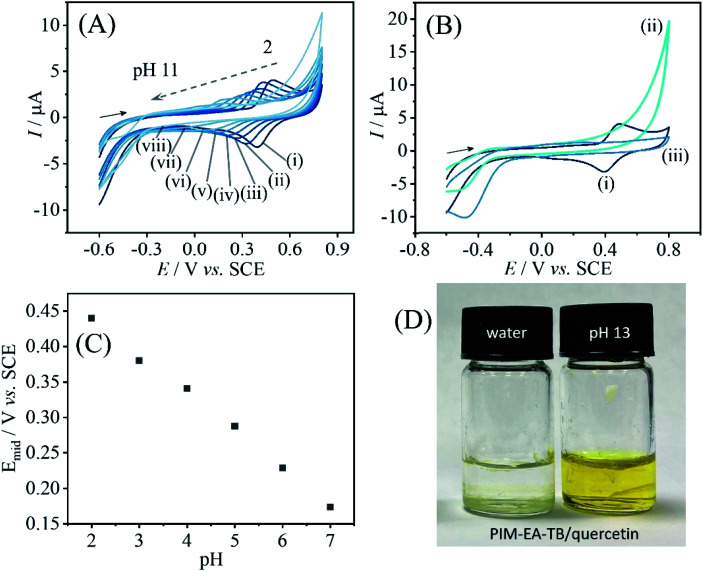
(A) Cyclic voltammograms (3 mm diameter glassy carbon electrode; scan rate 0.1 V s^−1^) for quercetin co-deposited into a PIM-EA-TB film and immersed into 0.1 M phosphate buffer at different pH values (pH = (i) 2, (ii) 3, (iii) 4, (iv) 5, (v) 6, (vi) 7, (vii) 9, and (vii) 11). (B) As before, but comparing pH 2, then pH 13, then back to pH 2. (C) Plot of midpoint potential *E*_mid_ = ½(*E*_p,ox_ + *E*_p,red_) *versus* pH. (D) Photograph showing the PIM-EA-TB/quercetin 1 : 1 film in a glass vial exposed to water and to 0.1 M phosphate buffer pH 13.

Data in Fig. 8A show the effect of solution pH on the voltammetric response for immobilised quercetin. A Nernstian shift in midpoint potential very similar to that observed for catechin is detected over a pH range from 2 to 7 (Fig. 8C). Data from more alkaline solution suggest leaching of quercetin. Fig. 8B shows data obtained subsequently for pH 2, then pH 13, then back in pH 2 and the signal for quercetin is clearly lost after exposure to alkaline conditions. When depositing PIM-EA-TB/quercetin into glass vials and adding water or phosphate buffer pH 13, the characteristic colour change for catechin anions released into the alkaline solution is observed (Fig. 8D).

### 3.4. Catechin and quercetin immobilisation by codeposition with PIM-EA-TB followed by electrochemically driven release

The release of the guest molecule from PIM-EA-TB occurs diffusion controlled and can be observed as a gradual loss of the voltammetric signal, slighter faster for the more water soluble catechin and somewhat slower for the less water-soluble quercetin. In order to quantify the release of these guest molecules, quantitative analysis of the solution phase by mass spectroscopy coupled to liquid chromatography was employed.

**Fig. 9:**
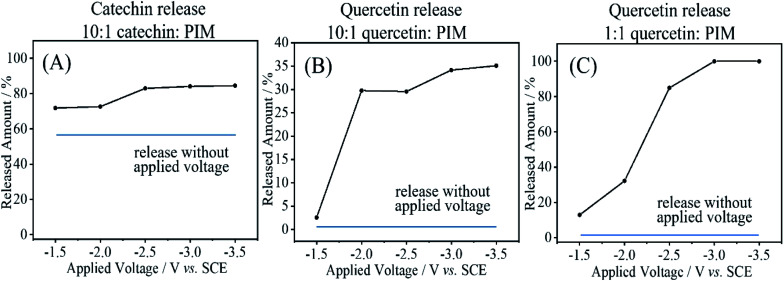
LC/MS data for the spontaneous and the electrochemically driven release of catechin (A) and quercetin (B and C). Blue line: data without electrochemical stimulation but for the same reaction time. Black line: release data obtained with electrochemical stimulation.

For catechin as 10 : 1 guest : host codeposit, the release into 10 mM phosphate buffer pH 7 was significant already over a period of 5 minutes (see Fig. 9A). Essentially most of the catechin is released within 5 minutes either with applied voltage or without. The applied voltage enhances the release by driving out the remaining 1 : 1 guest : host complex. Some remaining catechin is likely to be trapped in PIM-EA-TB outside of the zone of the glassy carbon electrode. For quercetin as 10 : 1 guest : host codeposit, the diffusion-driven loss is less dramatic. Fig. 9B shows data for no applied voltage where only less than 5% are released within 10 minutes. An applied negative voltage was then employed to locally create alkaline conditions at the electrode surface (associated with hydrogen evolution). It can be observed that at −2 V *vs.* SCE the process is effective and partial release is observed for the 1 : 10 host monomer : quercetin film. The amount of material released (approx. 35%) appears to be less than that expected based on the total loading. In part this could be due to the low solubility of quercetin preventing detection. Fig. 9C shows the case of release from a 1 : 1 host polymer repeat unit : quercetin film and close to 100% release is observed at potentials negative of −2.5 V *vs.* SCE. Electrochemical release can be effective for quercetin, but slow non-electrochemical release from the PIM-EA-TB micropores (for catechin fast) is always observed down to the level of the 1 : 1 host : guest system.

## 4. Conclusion

It has been shown that catechin or quercetin can effectively bind into PIM-EA-TB most likely *via* hydrogen bonding to tertiary amine sites. Alternatively, these molecular guests and polymer host can be co-deposited from solution in 1 : 1 stoichiometry to give films with essentially the same electrochemical properties. Accumulation of catechin occurs over a pH range from 2 to 7 irrespective of PIM-EA-TB protonation. Electrochemically driven release of both catechin or quercetin can be triggered at more alkaline pH values generated when applying sufficiently negative potentials. Electrochemical release of quercetin has been demonstrated by application of a negative applied potential (typically −2 V *vs.* SCE) to trigger a localised pH increase at the electrode surface. The key results of this work can be summarised as follows:

• PIM-EA-TB as microporous host can provide conditions for catechin or quercetin to accumulate and to be reversibly oxidised to the corresponding quinones. The electrochemical processes happen only in a very thin layer close to the carbon electrode surface (within tunnelling distance).

• Highly chemically reversible voltammetric responses suggest that there is no polymerisation or coupling of radical intermediates in the micropores of the PIM-EA-TB. This is consistent with limited mobility/reactivity of guests in the micropores.

• Binding of catechin into PIM-EA-TB is spontaneous at pH 2 (with protonation) or at pH 6 (without protonation) with similar binding constants and this can be attributed, at least in part, to hydrogen bonding interactions. Formation of 1 : 1 host polymer repeat unit : guest product are observed.

• Codeposition of PIM-EA-TB with catechin or quercetin is possible in any host monomer : guest ratio, but stable cyclic voltammetry responses are observed only for 1 : 1 ratios or lower guest concentrations. Excess guest species are readily released into the electrolyte solution.

• Electrochemically triggered release is effective for quercetin codeposits whereas catechin is more water soluble and therefore released at a higher rate without electrochemical stimulus.

In the future rigid polymer host materials such as PIMs could provide functional environments for accumulation and release of guests. The range of possible structural motifs is considerable and pore size as well as host–guest interactions could be tuned. Reactivity of PIM-EA-TB in aqueous acids may be associated with additional temporary ring-opening processes at the methano-bridge^38^ and therefore further study and comparison to the behaviour of other types of PIMs will be of interest. The ability to bind and release guests will be linked to functional properties (molecular interactions) as well as to pore size distribution/shape and could be further optimised for particular applications.

## Conflicts of interest

There are no conflicts to declare.

## Supplementary Material
